# Traumatic Brain Injury Diminishes Feedforward Activation of Parvalbumin-Expressing Interneurons in the Dentate Gyrus

**DOI:** 10.1523/ENEURO.0195-19.2020

**Published:** 2020-11-12

**Authors:** Kaitlin A. Folweiler, Guoxiang Xiong, Kaitlin M. Best, Hannah E. Metheny, Gabriel Nah, Akiva S. Cohen

**Affiliations:** 1 Department of Anesthesiology and Critical Care Medicine, Children’s Hospital of Philadelphia, Philadelphia, PA 19104; 2 Department of Anesthesiology and Critical Care, Perelman School of Medicine, University of Pennsylvania, Philadelphia, PA 19104; 3 Neuroscience Graduate Group, University of Pennsylvania, Philadelphia, PA 19104

**Keywords:** basket cells, dentate gyrus, hippocampus, interneurons, parvalbumin, traumatic brain injury

## Abstract

Traumatic brain injury (TBI) is associated with aberrant network hyperexcitability in the dentate gyrus (DG). GABA_A_ergic parvalbumin-expressing interneurons (PV-INs) in the DG regulate network excitability with strong, perisomatic inhibition, although the posttraumatic effects on PV-IN function after TBI are not well understood. In this study, we investigated physiological alterations in PV-INs one week after mild lateral fluid percussion injury (LFPI) in mice. PV-IN cell loss was observed in the dentate hilus after LFPI, with surviving PV-INs showing no change in intrinsic membrane properties. Whole-cell voltage clamp recordings in PV-INs revealed alterations in both EPSCs and IPSCs (EPSCs/IPSCs). Evoked EPSCs (eEPSCs) in PV-INs from perforant path electrical stimulation were diminished after injury but could be recovered with application of a GABA_A_-receptor antagonist. Furthermore, current-clamp recordings using minimal perforant path stimulation demonstrated a decrease in evoked PV-IN action potentials (APs) after LFPI, which could be restored by blocking GABA_A_ergic inhibition. Together, these findings suggest that injury alters synaptic input onto PV-INs, resulting in a net inhibitory effect that reduces feedforward PV-IN activation in the DG. Decreased PV-IN activation suggests a potential mechanism of DG network hyperexcitability contributing to hippocampal dysfunction after TBI.

## Significance Statement

Traumatic brain injury (TBI) damages the hippocampus and causes long-lasting memory deficits. After TBI, the dentate gyrus (DG), a crucial regulator of cortical input to the hippocampus, undergoes a dysfunctional net increase in excitation, although the circuit mechanisms underlying this network excitatory-inhibitory (E/I) imbalance are unclear. In this study, we found that TBI alters synaptic inputs onto an inhibitory interneuron population [parvalbumin (PV)-INs) in the DG which results in the decreased firing activity of these neurons because of a net inhibitory influence. The inhibition of PV-INs demonstrates a potential mechanism contributing to DG network hyperexcitability and hippocampal dysfunction after TBI.

## Introduction

Fast-spiking, parvalbumin-expressing GABAergic interneurons (PV-INs) are powerful regulators of excitability in neural networks and play an important role in mediating hippocampal-dependent cognitive behaviors (Freund and Buzsáki, 1996; Nitz and McNaughton, 2004; Fuchs et al., 2007; Armstrong and Soltesz, 2012; Hu et al., 2014). In the hippocampus, PV-INs contribute to the ability of the dentate gyrus (DG) subregion to act as a filter or gate of incoming sensory information from the cortex by providing strong feedforward inhibition onto granule cells (Coulter and Carlson, 2007). In combination with the low intrinsic membrane excitability of granule cells, PV-IN GABAergic inhibition contributes to sparse granule cell action potential (AP) firing under normal conditions (Ewell and Jones, 2010; Kraushaar and Jonas, 2000).

After traumatic brain injury (TBI), the DG experiences network hyperexcitability (Lowenstein et al., 1992; Toth et al., 1997; Santhakumar et al., 2000; Witgen et al., 2005). Granule cells no longer sparsely fire APs, and evoked extracellular burst discharges are increased in the granule cell layer *in vivo* (Lowenstein et al., 1992). This shift toward a hyperexcitable network state leads to a break down in the physiological filtering function of the DG and is associated with spatial memory impairments (Folweiler et al., 2018).

One week after TBI, the frequency of miniature IPSCs (mIPSCs) is reduced in granule cells, suggesting that a loss of synaptic inhibition is contributing to granule cell hyperexcitability (Toth et al., 1997; Witgen et al., 2005). While previous studies have looked at other populations of dentate inhibitory interneurons after TBI (Hunt et al., 2011; Butler et al., 2017), the potential role of PV-INs in dentate network hyperexcitability after injury has yet to be examined. In order to understand the effects of TBI on PV-IN inhibition, we investigated the intrinsic membrane properties and synaptic inputs of PV-INs in the DG one week after mild lateral fluid percussion injury (LFPI).

## Materials and Methods

### Mice

All experiments were performed in accordance with protocols approved by our institution’s Institutional Animal Care and Use Committee and the guidelines established by the United States Public Health Service’s Guide for the Care and Use of Laboratory Animals. Experiments were designed to minimize the number of animals required and those used were cared for, handled, and medicated as appropriate to minimize their suffering. To visually identify PV-INs, PV^CRE^ transgenic mice, expressing Cre-recombinase in PV-expressing neurons (129P2^Pvalbtm1(cre)Arbr^/J; The Jackson Laboratory; RRID:IMSR_JAX:008069) were crossed with tdTomato reporter mice (129S6-Gt(ROSA)26Sor*^m14(CAG-tdTomato)Hze^*/J; The Jackson Laboratory, RRID:IMSR_JAX:007908) to generate PV^CRE^;*tdTomato*+/− (i.e., PV-Tomato) transgenic animals which express tdTomato fluorescence in parvalbumin-positive (PV+) cells. All experiments were performed on six- to eight-week-old male and female PV-Tomato mice. The primary purpose of using both male and female mice was to use all transgenic animals that were bred for experiments with the secondary aim of reducing sex bias by favoring one sex over another (Will et al., 2017). The number of male and female mice in each group is listed by experiment in Table 1.

**Table 1:** Male and female mice per group used in each experiment

	Sham		LFPI	
Experiment	Male (*n*)	Female (*n*)	Male (*n*)	Female (*n*)
Immunostaining	3	0	4	0
Cell counts	6	2	4	5
Timm staining	10	0	10	0
fEPSPs	4	2	3	3
Intrinsic properties	5	5	4	6
mEPSCs	4	3	3	3
eEPSCs	7	5	8	3
mIPSCs	2	4	5	2
Evoked APs	3	2	3	2

For each experiment (left column) the sample size of male and female mice in sham and LFPI groups. *n*, sample size.

### Surgical procedures

Animals were anesthetized with a mixture of ketamine (2.6 mg/kg) and xylazine (0.16 mg/kg) via intraperitoneal injection. Once fully anesthetized, animals were placed in a stereotaxic frame (Stoetling), the scalp was incised and pulled away to fully expose the right parietal bone. An ultrathin Teflon disk, with the outer diameter equal to the inner diameter of a trephine was glued to the skull with Vetbond (3 M) between lambda and bregma sutures, and between the sagittal suture and the lateral ridge over the right hemisphere. Guided by the Teflon disk, a trephine was used to perform a 3-mm diameter craniectomy over the right parietal area. Following craniectomy, a Luer-lock needle hub (3-mm inner diameter) was secured above the skull opening with superglue (Loctite) and dental acrylic, filled with saline and capped. Lastly, animals were removed from stereotaxis, placed on a heating pad until fully recovered from anesthesia, and then returned to their respective home cage.

### LFPI

Twenty-four hours after craniectomy, animals were placed under isoflurane anesthesia (2% O_2_ in 500 ml/min) in a chamber and respiration was visually monitored until animals reached a surgical plane of anesthesia (one respiration per 2 s). At this point, animals were removed from isoflurane, the needle hub was refilled with saline and connected to the fluid percussion injury device (Department of Biomedical Engineering, Virginia Commonwealth University, Richmond, VA) via high-pressure tubing. The animal was placed onto a heating pad on its left side and on resumption of normal breathing pattern but before sensitivity to stimulation, the injury was induced by a 20-ms pulse of saline onto the intact dura. The pressure transduced onto the dura was monitored with an oscilloscope, with injury severity ranging between 1.4 and 1.6 atmospheres (atm). Immediately after injury, the hub was removed from the skull and the animal was placed in a supine position to assess righting reflex. After righting, the animal was subjected to inhaled isoflurane to suture the scalp. Animals were allowed to recover on a heating pad until mobile, at which point they were returned to their home cage. Sham animals underwent all surgical procedures including attachment to the FPI device with exclusion of the actual fluid pulse.

### PV-IN cell counting

**Figure 1.**
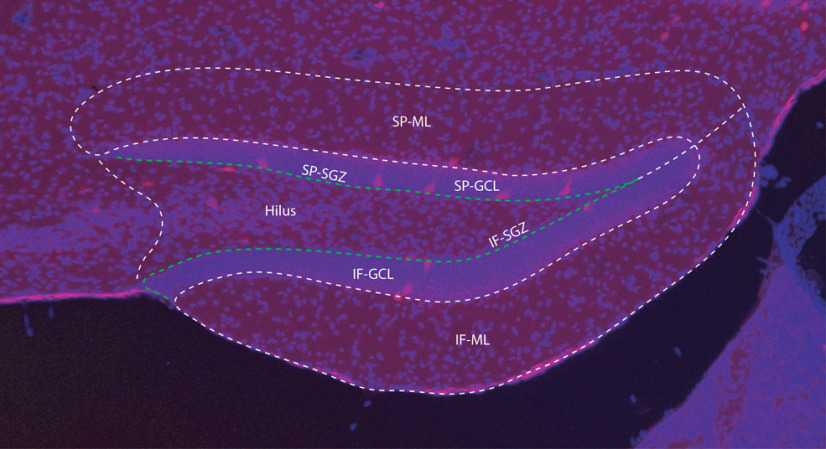
Anatomical layers of the DG used to determine PV+ cell location for counting. Overlay of Hoechst staining (blue) of all cells in the slice was used to identify the anatomic structure and cellular layer boundaries (dotted white line) of the DG when counting tdTomato-expressing (red) PV+ cells. Cell anatomic location was divided by both dentate blade, suprapyramidal (SP) or infrapyramidal (IF), and cellular layer, including molecular layer (ML), granule cell layer (GCL), subgranular zone (SGZ), and hilus. TdTomato-expressing cells in the hilus but within one soma length (20–30 μm) of the granule cell layer were considered in the subgranular zone (white arrow). Scale bar (lower left): 50 μm.

To show changes (if any) in number of PV-Tomato interneurons resulted from TBI, sham and LFPI mice (7 d after injury) were deeply anesthetized with 5% chloral hydrate and perfused with 15 ml of saline followed by 50 ml of paraformaldehyde (4% in phosphate buffer, pH 7.4; Sigma-Aldrich). The brains were removed and postfixed in the fixative for 90 min. Vibratome slices were cut at 50 μm in thickness with a VT 1000S (Leica) and collected in serial from a fixed brain. The slices were treated with 0.3% Triton X-100 for 1 h at room temperature (RT) and then counterstained with Hoechst, a DNA dye to stain nuclei of all cells in a slice. Hoechst staining (blue) made it easier to outline the structure and boundary of the hippocampus when counting PV-Tomato cells (red) in DG (Fig. 1). Fluorescent images were acquired with an Olympus BX-51 microscope at 10× magnification. All tdTomato-positive neurons in the DG ipsilateral to the injury site were quantified using modified stereology with the optical fractionator method in which every sixth section through the rostral/caudal extent of the dorsal hippocampus (bregma −1.00 to −2.75 mm) was examined (West et al., 1991; Eisch et al., 2000; Mouton, 2002). Cells were scored as tdTomato-positive if tdTomato labeling in the soma was more intense than background (Lim et al., 2013). Since counting of cells was conducted on every sixth section of the hippocampus, the number of cells in each anatomic region was multiplied by six to obtain the reported estimate of the total number of cells per region. Cell anatomic location was considered by both dentate blade (suprapyramidal or infrapyramidal) and cellular layer, including molecular layer, granule cell layer, subgranular zone, and hilus. TdTomato-positive cells in the hilus but within one soma length (20–30 μm) of the granule cell layer were considered in the subgranular zone (Fig. 1).

### Immunofluorescent staining

**Figure 2.**
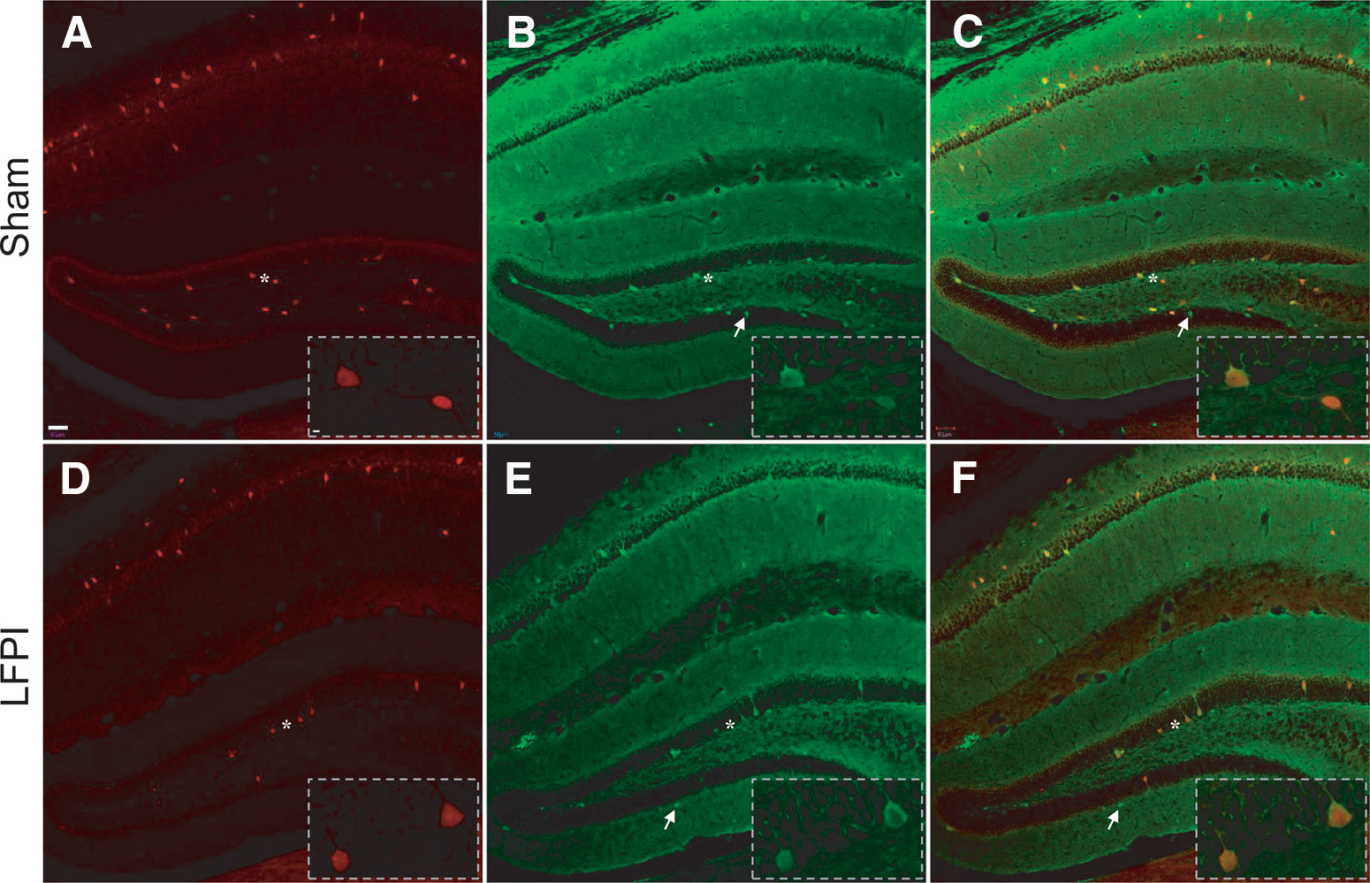
Immunofluorescent staining to confirm dentate PV expression in tdTomato-positive cells in both sham (top row) and LFPI (bottom row) animals 7 d after surgery. ***A***, ***D***, Transgenic expression of tdTomato fluorescence (red) in PV+ cells expressing Cre-recombinase. ***B***, ***E***, Immunofluorescent staining of PV-expressing cells (green). ***C***, ***F***, Co-localization of td-Tomato and immunofluorescence (inset) demonstrates that most td-Tomato-expressing cells are PV+. Asterisk indicates the location of the inset. A small portion of PV-expressing cells (green) did not express td-Tomato (white arrows).

To show whether PV-Tomato cells in DG from the transgenic mouse did express PV, we also performed PV immunofluorescent staining on brain slices containing the hippocampus from sham and injured mice. The slices were treated with 0.3% Triton X-100 and blocked with a mixture of 1% BSA and 5% normal goat serum, 60 min, respectively. They were then incubated with a monoclonal antibody against PV (1:1000; Sigma-Aldrich), 60 min at RT and overnight at 4°C. Visualization was done by incubating the slices with Alexa Fluor 488-conjugated goat anti-mouse IgG for 75 min at RT. The immunostained slices were mounted on precleaned slide glass and coverslipped with aqueous mounting medium. In a stained brain slice, PV-immunostained cells showed green and PV-Tomato cells red (Fig. 2). PV-Tomato cells expressing PV should be identified as yellowish to brown, depending on the intensity of tdTomato (red).

### Timm staining

To examine whether mossy fiber spouting existed in the hippocampus, Timm staining was performed according to a protocol reported previously (Van der Zee et al., 1995). Mice were perfused with 15 ml of saline followed by 50 ml of sodium sulfide perfusion medium containing 8.9 g of Na_2_S.9H_2_O, 10.9 g of sucrose and 1.19 g of Na_2_PO4.H_2_O dissolved in 100 ml of deionized water (DW). The brains were removed and postfixed in the same perfusion medium for 3 h at RT. Untreated vibratome slices (50 μm in thickness) were mounted on gelatin-coated slide glass and air-dried overnight. They were then incubated in dark for 60 min with a mixture containing 6 volume of gum arabic (50 g/100 ml), 3 volume of hydroquinone (5.67 g/100 ml), and 1 volume of citric acid-sodium citrate buffer (25.5 and 23.5 g, respectively, in a total of 100 ml). For each 100 ml of the incubation solution, 0.5 ml of silver nitrate stock solution (1.7 g AgNO_3_/10 ml) was added. Staining was stopped by brief rinse in DW three times. The slices (on glass) were then dehydrated and cleared before coverslipped with Permount. Brightfield microscope was used to observe positive Timm staining. Mossy fiber sprouting (MFS) was defined as Timm-stained axonal collaterals (black) present in the molecular layer of DG.

### Electrophysiology

All recordings were made 5–9 d after LFPI or sham surgery. Mice were anesthetized with isoflurane, and the brains were quickly and carefully removed, then placed into ice-cold oxygenated (95% O_2_/5% CO_2_) sucrose artificial CSF (ACSF) containing the following: 202 mm sucrose, 3 mm KCl, 1.25 mm NaH_2_PO_4_, 26 mm NaHCO_3_, 10 mm glucose, 1 mm MgCl_2_, and 2 mm CaCl_2_. Coronal slices 350 μm thick containing the dorsal hippocampus were cut on a VT1200S vibratome (Leica Microsystems Inc.) and transferred to 33–37°C normal ACSF containing the following: 130 mm NaCl, 3 mm KCl, 1.25 mm NaH_2_PO_4_, 26 mm NaHCO_3_, 10 mm glucose, 1 mm MgCl_2_, and 2 mm CaCl_2_, for at least 45 min.

### Extracellular field recordings

Electrodes for recordings field EPSPs (fEPSPs) were fabricated from borosilicate glass (World Precision Instruments, #1B150F-4), pulled to a tip resistance of 2–6 MΩ and filled with ACSF. fEPSPs were recorded from an electrode placed in the suprapyramidal molecular layer. Stimulating electrodes were non-concentric bipolar (World Precision Instruments, #ME12206) and placed at the apex of the molecular layer. Electrical stimuli were 100 μs in duration. Field potential input/output (I/O) relationships were recorded in response to increasing stimulating intensities (20- to 400-μA stimulation, 20-μA increments, 8-s interstimulus interval) from each slice. For each stimulation intensity, recordings were averaged from three trials, and the fEPSP slope was calculated for the first linear portion of the fEPSP (i.e., monosynaptic response). Recordings were obtained with an Axoclamp 900A amplifier and pClamp10 data acquisition software (Molecular Devices; RRID:SCR_011323), filtered at 2 kHz. Field potential data were analyzed using pClamp10 software and custom-written scripts in MATLAB R2012b (MathWorks).

### Whole-cell patch clamp recordings

Patch electrodes with resistances of 4–7 MΩ were pulled from borosilicate glass (World Precision Instruments). Series resistance was monitored throughout the experiment and recordings were discontinued if series resistance exceeded 25 MΩ at any point. Series resistance was compensated for at 70–80% compensation. All recordings were made using a Multiclamp 700B (Molecular Devices) sampled at 20 kHz, filtered at 2 kHz. Electrophysiological data were analyzed using Clampfit 10 and MATLAB software. Synaptic events were determined via the Template Search algorithm in Clampfit 10.

PV-INs were visually identified by fluorescent tdTomato expression in cells within the granule cell layer, subgranular zone, or hilar subregion within 100 μm of the hilar-granule cell layer interface. In order to confirm that tdTomato-expressing neurons also had a fast-spiking electrophysiological signature, experiments began with a series of depolarizing current steps. Any neurons that failed to demonstrate non-accommodating trains of APs with a maximum spiking frequency >30 Hz (Kawaguchi and Kubota, 1997) in response to depolarizing current injections, or that demonstrated baseline instability, were excluded from further analysis. All whole-cell recordings were performed in one cell per brain slice and are reported as n cells per n mice in each group.

Resting membrane potential was computed as the average voltage in the first 2 s immediately after whole-cell configuration was achieved. All other intrinsic excitability measures were computed from current clamp recordings consisting of a series of 10 500-ms current steps, from −100 to 250 pA in 50-pA increments. Constant holding current was applied to maintain the neuron at −65 mV before and after current steps. AP threshold was computed by taking dV/dt of the voltage trace at 175 pA in the intrinsic excitability experiments and then averaging the corresponding voltage values for the first 10 spikes where dV/dt exceeded 30 mV/ms (Howard et al., 2007). Input resistance was determined from the steady-state voltage response for the four initial current steps (−100 to 50 pA). AP frequency and corresponding interspike intervals were calculated for all current steps resulting in AP firing.

For whole-cell patch-clamp recording of EPSCs and intrinsic excitability measures electrode internal solution contained the following: 140 mm K-gluconate, 5 mm EGTA, 10 mm HEPES, 1 mm MgCl_2_, 1 mm CaCl_2_, 3 mm KOH, and 2 mm Mg-ATP and was titrated to a final pH of 7.1–7.3 with KOH and osmolality of 290–300 mOsm. Bicuculline methiodide (BMI; 30 μm, Abcam) was added before voltage clamp recordings, and tetrodotoxin (TTX; 0.4 μm, Abcam) was added to isolate miniature events (mEPSCs). Neurons were voltage-clamped at −65 mV for all EPSC voltage-clamp experiments. Liquid junction potential of 16.9 mV (calculated in Clampex) was corrected for in all data reported from these experiments. For whole-cell patch-clamp recording of inhibitory currents, internal solution contained the following: 140 mm Cs-gluconate, 1 mm NaCl, 5 mm EGTA, 1 mm MgCl_2_, 1 mm CaCl_2_, 3 mm KOH, and 2 mm Mg-ATP and was titrated to a final pH of 7.2–7.3 with CsOH and an osmolality of 290–300 mOsm. APV (50 μm, Abcam), CNQX (6 μm, Abcam), and QX-314 (5 mm, Abcam) were added before voltage clamp recordings, and TTX (0.4 μm) was added to isolate mIPSCs. Liquid junction potential of 2.0 mV was corrected for in all data reported from these experiments. Neurons were voltage-clamped at 0 mV for all voltage clamp experiments of inhibitory currents. The slice chamber temperature for all recordings was set to 29–31°C.

To record perforant path-evoked EPSCs (eEPSCs), a non-concentric bipolar placed in at the apex of the molecular layer was used to stimulate afferent axons onto patch-clamped PV-INs held at −65 mV in voltage-clamp. Evoked potentials were recorded in response to electrical stimulation from 10 to 100 μA in 10-μA incremental steps). Three I/O eEPSCs were recorded and averaged from each cell. Extracellular fEPSPs were simultaneously recorded in the molecular layer during evoked voltage-clamp and current-clamp experiments and eEPSC for each recorded cell were normalized to the fiber volley amplitude of the slice. As for fEPSP recordings, the first linear, monosynaptic portion of the eEPSC was used to calculate the slope. For current-clamp experiments, an electrode was placed in the perforant path at the apex of the molecular layer. Minimal current stimulation was adjusted to find the lowest current intensity parameter that could elicit at least one AP in a series of 20 stimulations. Once the minimal stimulation current intensity was set, three series of 20 stimulations (12-s interstimulus interval) were administered and the number of stimulus-evoked APs for each series was recorded and reported as a percentage of the total number of stimulations in that series (20 stimulations per series). Throughout current-clamp recordings, a slow injection of current was given to maintain a membrane potential of −65 mV. Stimulus-evoked APs were recorded in normal ACSF baseline and then in the presence of 100 nm picrotoxin (PCTX). Cells were recorded in voltage clamp after PCTX wash-in to confirm that IPSCs were eliminated.

### Statistical procedures

A priori power calculations using β = 0.8 were performed using G*Power statistical software to determine the minimum sample size required for each experiment (Faul et al., 2007). Electrophysiological and cell count data were analyzed using pClamp 10 and GraphPad Prism 7.0 software (GraphPad Software; RRID:SCR_002798). Data distributions were initially tested for normality using both Shapiro–Wilk and D’Agostino–Pearson tests. Statistics were performed using either unpaired Student’s *t* test, or two-way repeated measures ANOVA with Sidak’s multiple comparison test unless use of another test is indicated (Table 2). In experiments with small sample sizes, effect sizes were reported using Glass’s *d*. Cumulative distribution functions of synaptic event properties were constructed by randomly sampling the same number of events (*n* = 75) from each cell. The Kolmogorov–Smirnov (K-S) test was used for statistical comparison of synaptic current measurements. *N* refers to number of cells and number of animals as described in the results of each experiment. Statistical significance was set at *p* < 0.05.

**Table 2:** Statistical analysis of experimental results

	Experiment	Data structure	Type of test	Test statistic	*p* value
Immunostaining					
1a	Td-Tomato/PV-immunostain colocalization	Normal (SW and DP)	Two-tailed unpaired *t* test	*t* = 0.065 df = 4.42	*p* = 0.95
1b	PV-immunostained cells only	Normal (SW and DP)	Two-tailed unpaired *t* test	*t* = 0.721 df = 5	*p* = 0.47
1c	Td-Tomato cells only	Normal (SW and DP)	Two-tailed unpaired *t* test	*t* = 0.774 df = 5	*p* = 0.56
Cell counts					
2a	Total DG	Normal (SW and DP)	Two-tailed unpaired *t* test	*t* = 1.971 df = 15	*p* = 0.07
2b	Hilus	Normal (SW and DP)	Two-tailed unpaired *t* test	*t* = 2.431 df = 15	*p* = 0.03
2c	Subgranular zone	Normal (SW and DP)	Two-tailed unpaired *t* test	*t* = 1.579 df = 15	*p* = 0.14
2d	Granule cell layer	Normal (SW)	Two-tailed unpaired *t* test	*t* = 0.6157 df = 15	*p* = 0.55
2e	Molecular layer	Normal (SW and DP)	Two-tailed unpaired *t* test	*t* = 0.9811 df = 15	*p* = 0.34
2f	Suprapyramidal blade	Normal (SW and DP)	Two-tailed unpaired *t* test	*t* = 2.048 df = 15	*p* = 0.06
2g	Infrapyramidal blade	Normal (SW and DP)	Two-tailed unpaired *t* test	*t* = 1.117 df = 15	*p* = 0.28
2f	Septotemporal	Normal (DP)	Two-way ANOVA	*F*_(1,87)_ = 0.87	*p* = 0.35
Intrinsic properties					
3a	fEPSPs	Normal (SW and DP)	Repeated measures ANOVA	*F*_(19,190)_ = 14.34	*p* < 0.0001
3b	Input resistance	Normal (DP)	Two-tailed unpaired *t* test	*t* = 0.3717 df = 18	*p* = 0.71
3c	Resting membrane potential	Normal (SW and DP)	Two-tailed unpaired *t* test	*t* = 1.279 df = 18	*p* = 0.21
3d	AP threshold	Normal (SW)	Two-tailed unpaired *t* test	*t* = 1.244 df = 18	*p* = 0.23
3e	AP firing frequency	Normal (SW)	Repeated measures ANOVA	*F*_(1,13)_ = 0.422	*p* = 0.53
3f	AP half-width	Normal (SW)	Two-tailed unpaired *t* test	*t* = 0.612 df = 14	*p* = 0.55
mEPSCs					
4a	Interevent interval	Non-normal (SW and DP)	K-S test	*D* = 0.162	*p* < 0.0001
4b	Amplitude	Non-normal (SW and DP)	K-S test	*D* = 0.113	*p* = 0.006
4c	Rise τ	Non-normal (SW and DP)	K-S test	*D* = 0.104	*p* = 0.004
4d	Decay τ	Non-normal (SW and DP)	K-S test	*D* = 0.072	*p* = 0.20
eEPSCs					
5a	Slope	Normal (SW and DP)	Two-way ANOVA	*F*_(1,13)_ = 0.1825	*p* = 0.68
5b	Amplitude	Normal (SW and DP)	Two-way ANOVA	*F*_(1,22)_ = 4.715	*p* = 0.04
5c	Charge transfer	Normal (SW and DP)	Two-way ANOVA	*F*_(1,21)_ = 5.426	*p* = 0.03
5d	Slope (BMI wash-in)	Normal (SW and DP)	Two-way ANOVA	*F*_(1,10)_ = 0.005518	*p* = 0.94
5e	Amplitude (BMI wash-in)	Normal (SW and DP)	Two-way ANOVA	*F*_(1,10)_ = 0.8552	*p* = 0.38
5f	Charge transfer (BMI wash-in)	Normal (SW and DP)	Two-way ANOVA	*F*_(1,10)_ = 0.9528	*p* = 0.35
mIPSCs					
6a	Amplitude	Non-normal (SW and DP)	K-S test	*D* = 0.134	*p* = 0.0003
6b	Rise τ	Non-normal (SW and DP)	K-S test	*D* = 0.126	*p* = 0.008
6c	Interevent interval	Non-normal (SW and DP)	K-S test	*D* = 0.064	*p* = 0.25
6d	Decay τ	Non-normal (SW and DP)	K-S test	*D* = 0.038	*p* = 0.86
Evoked APs					
7a	Resting membrane potential	Normal (SW)	Welch’s unpaired *t* test	*t* = 0.9494 df = 8	*p* = 0.37
7b	Whole-cell capacitance	Normal (SW)	Welch’s unpaired *t* test	*t* = 0.4834 df = 8	*p* = 0.64
7c	Evoked APs (normal ACSF)	Normal (SW)	Welch’s unpaired *t* test	*t* = 9.084 df = 4	*p* = 0.0008
7d	Evoked APs (sham pre-PCTX and post-PCTX)	Normal (SW)	Paired *t* test	*t* = 11.27, df = 4	*p* = 0.0004
7e	Evoked APs (LFPI pre-PCTX and post-PCTX)	Normal (SW)	Paired *t* test	*t* = 5.119 df = 4	*p* = 0.0034
9f	Evoked APs (100 nm PCTX)	Normal (SW)	Welch’s unpaired *t* test	*t* = 1.138 df = 6	*p* = 0.29

For each experiment (second column), the statistical results included the structure of the data distribution as determined by Shapiro–Wilk (SW) or D’Agostino–Pearson (DP) normality tests (third column), the statistical test used, the test statistic and degrees of freedom (df), and the *p* value; *p* values >0.05 are rounded up to two decimal places. DG, dentate gyrus; PCTX, picrotoxin.

## Results

### PV immunostaining confirms td-Tomato expression in PV+ cells

Cre-tdTomato colocalization was confirmed in a subset of animals (sham *n* = 3, LFPI *n* = 4). Cells were classified as having tdTomato and PV-immunostaining colocalization, PV-immunostaining only, or tdTomato only (Fig. 2). There was no difference in the number of cells where tdTomato and PV-immunostaining overlapped (sham: mean ± SD = 78.4 ± 5.5% of total cells; LFPI: mean ± SD = 78.9 ± 11.9% of total cells, *p* = 0.949; Table 2, a). The majority of cells that did not colocalize tdTomato and PV+, were PV+ only (sham = 18.5 ± 5.9%; LFPI = 13.8 ± 9.7% total cells), and there was no difference in the number of PV+ only cells between sham and LFPI animals (*p* = 0.47; Table 2, 1b). The occurrence of tdTomato only cells was extremely rare (sham = 3.0 ± 5.3%; LFPI = 0.99 ± 1.4%, *p* = 0.56; Table 2, 1c) and did not differ significantly between the injury groups. While tdTomato was not expressed in all PV+ cells, this analysis shows that almost all tdTomato cells were PV+ and counting tdTomato cells provides a good estimate for PV cell prevalence.

### Mild LFPI induces loss of hilar tdTomato-expressing neurons

**Figure 3.**
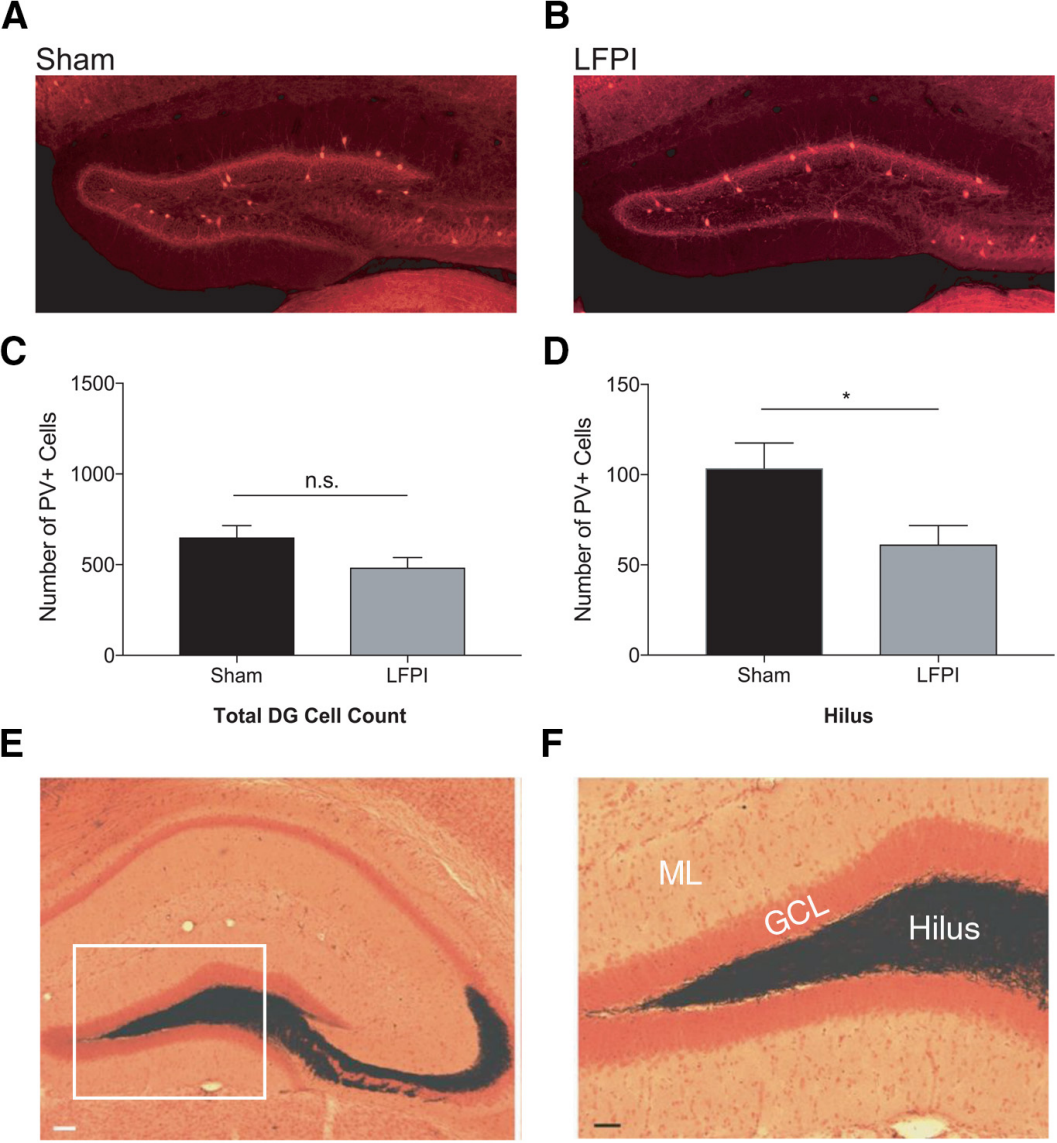
PV-IN cell loss in the dentate hilus occurs one week after mild LFPI. PV+ inhibitory interneurons expressing the fluorescent marker tdTomato in the DG one week after (***A***) sham surgery or (***B***) LFPI. ***C***, Total number of PV+ cell bodies in the DG are not significantly different between sham and LFPI. ***D***, The hilus experiences a significant loss of PV+ interneurons. n.s., non-significant; *p < 0.05. ***E***, Timm staining of granule cell mossy fiber projections (black) into the hilus and area CA3 in an LFPI animal 7 d after injury; scale bar: 50 μm. Inset (white box) magnified in ***F*** demonstrates no Timm-stained fibers are present in the inner molecular layer (ML) adjacent to the granule cell layer (GCL) where MFS would occur. Scale bar: 25 μm.

Previous studies have found fewer hilar GABA-expressing neurons, including PV-INs, one week after fluid percussion injury; however, these studies used a more moderate (2.0–2.2 atm) pressure impact than that implemented in this study (Lowenstein et al., 1992; Toth et al., 1997; Santhakumar et al., 2000; Hunt et al., 2011). Additionally, some studies have observed posttraumatic MFS in the DG molecular layer (Hunt et al., 2011). To test whether hilar PV-IN cell loss occurred in our mild (1.4–1.6 atm) LFPI model, we counted the number of tdTomato-expressing cells in the dorsal DG ipsilateral to the injury site in sham (*n* = 8 50-μm sections per mouse, 8 mice; Fig. 3*A*) and LFPI (*n* = 8 50-μm sections per mouse, 9 mice; Fig. 3*B*). At one week after injury, the total number of dentate PV-INs appeared to trend downward but did not result in a statistically significant change after injury (sham: mean ± SEM = 650 ± 65 cells; LFPI mean ± SEM = 485 ± 54, *p* = 0.067; Fig. 3*C*; Table 2, 2a). However, there were significantly less tdTomato-positive cells in the hilus (sham: mean ± SEM = 104 ± 14 cells; LFPI mean ± SEM = 61 ± 10, *p* = 0.028; Fig. 3*D*; Table 2, 2b). PV-IN cell counts remained unchanged in the subgranular zone, granule cell layer, molecular layer, suprapyramidal blade, and infrapyramidal blade when, respectively, counted (Table 2, 2c–2g). There were no septotemporal differences in PV-IN cell counts (two-way ANOVA, *F*_(1,87)_ = 0.87, *p* = 0.35; Table 2, 2f). Furthermore, to examine whether MFS was present in our LFPI model 7 d after injury, we performed Timm staining to label mossy fiber projections (i.e., granule axons and axon terminals) in the DG (*n* = 10 animals in each group). No Timm-stained fibers were observed in the molecular layer of LFPI (Fig. 3*E*,*F*) or sham animals at 7 d after injury, suggesting that MFS was not present at the time point observed after injury in our LFPI model.

### Dentate network hyperexcitability persists in PV-Tomato mouse line

**Figure 4.**
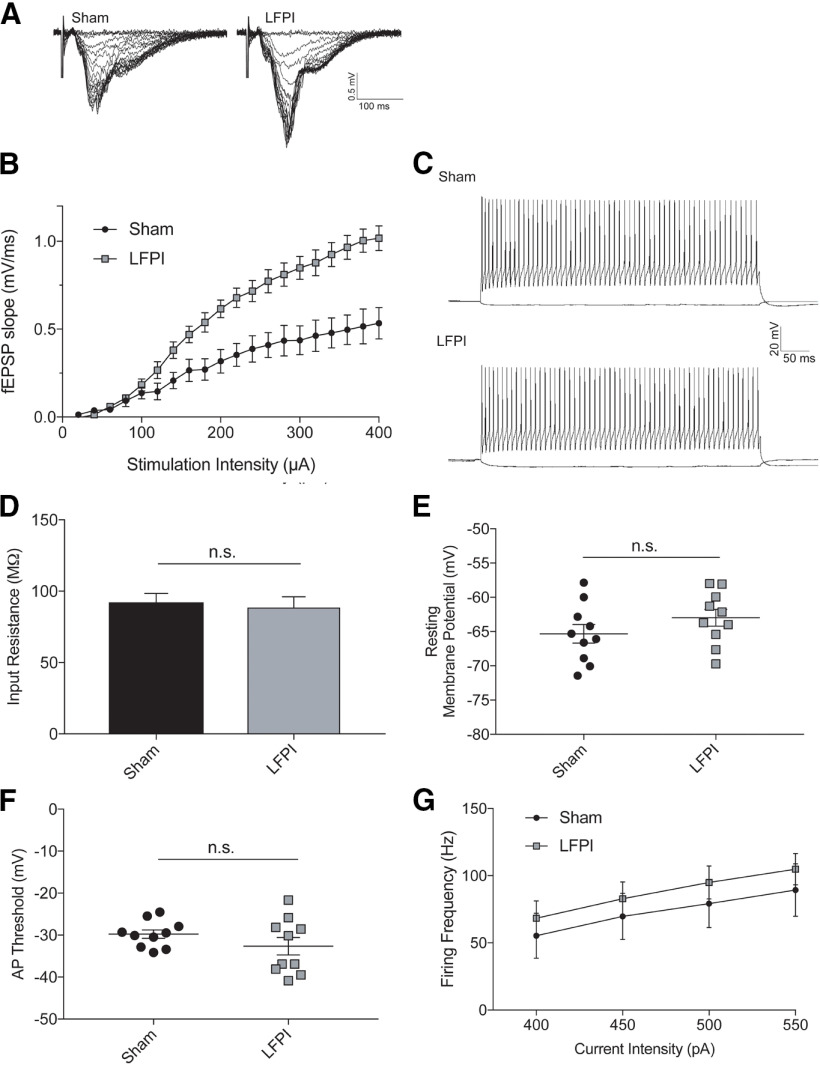
PV-IN intrinsic membrane excitability remains intact after LFPI. ***A***, fEPSPs recorded from dentate molecular layer suggests dentate network hyperexcitability in transgenic PV-Tomato mouse line in response to perforant path electrical stimulation as demonstrated by (***B***) significantly larger fEPSP slopes in LFPI mice than sham mice within the stimulation intensity range of 20–400 μA, 20-μA increments, 100-μs duration. ***C***, Example membrane voltage traces from sham (top trace) and LFPI (bottom trace) PV-INs in response to −50 pA and +100 pA current injections. Passive membrane properties of PV-INs are unchanged by injury as exemplified by (***D***) membrane input resistance and (***E***) resting membrane potential. Active firing properties also show no significant difference between sham (black points) and injured (gray points) on (***F***) AP threshold and (***G***) PV-IN firing frequency in response to increasing depolarizing current injections. n.s., non-significant.

To ensure that injury alters dentate excitability in our transgenic PV-Tomato mice, I/O curves were generated by electrically stimulating perforant path fibers (stimulation intensity range: 20–400 μA, 20-μA increments, 100-μs duration) and recording extracellular field potentials in the molecular layer one week after LFPI or sham surgery (*n* = 6 mice in each group, three slices per animal) with transgenic animals (Fig. 4*A*). In brain slices from sham animals, fEPSP slope increased almost linearly as perforant path stimulation intensity increased (Fig. 4*B*). Slices from LFPI animals demonstrated significantly larger fEPSP slopes with increasing stimulation intensity compared with sham (repeated measures ANOVA, *F*_(19,190)_ = 14.34; *p* < 0.0001; Table 2, 3a). This injury-induced shift in the I/O curve demonstrates that dentate posttraumatic hyperexcitability is present in the transgenic mouse line used for additional experiments in this study.

### PV-IN intrinsic membrane properties are unaffected by injury

Neuronal intrinsic membrane properties, dictated predominantly by membrane proteins and their subsequent activity, play a significant role in a neuron’s propensity to fire APs. After experimental TBI, alterations in intrinsic properties have been observed in dentate glutamatergic neurons (Howard et al., 2007; Gupta et al., 2012). To investigate the intrinsic properties of dentate PV-INs, we performed whole-cell patch clamp recordings one week after LFPI or sham surgery (*n* = 10 cells in each group, one cell per animal; Fig. 4*C–G*). Passive properties such as membrane input resistance (sham: 92.2 ± 6.3 MΩ; LFPI: 88.6 ± 7.5 MΩ; *p* = 0.71; Fig. 4*D*; Table 2, 3b), and resting membrane potential (mean ± SEM = −65.3 ± 1.4 mV in sham and −62.9 ± 1.2 mV in LFPI; *p* = 0.22; Fig. 4*E*; Table 2, 3c), were not significantly different between cells from sham and LFPI groups. Additionally, PV-IN active firing properties were not affected by injury. PV-INs from sham animals had on average an AP threshold of −29.8 ± 1.0 mV, while cells from LFPI animals had an average firing threshold of −32.7 ± 2.1 mV (*p* = 0.23; Fig. 4*F*; Table 2, 3d). AP firing frequency in response to depolarizing current steps (400–550 nA, 50-nA steps) was unaltered by injury (sham: *n* = 8 cells, LFPI: *n* = 7 cells; repeated measures ANOVA: *F*_(1,13)_ = 0.42; *p* = 0.53; Figs. 2*G*, 4*C*; Table 2, 3e). Lastly, AP half-width, was not significantly different between sham and injured groups (sham: mean ± SEM = 0.74 ± 0.06 ms; LFPI: mean ± SEM = 0.69 ± 0.04 ms; *p* = 0.55, Table 2, 3f). These findings suggest that there is no net change in either passive or active intrinsic membrane properties of PV-INs after injury.

### Excitatory synaptic inputs onto PV-INs exhibit posttraumatic changes

**Figure 5.**
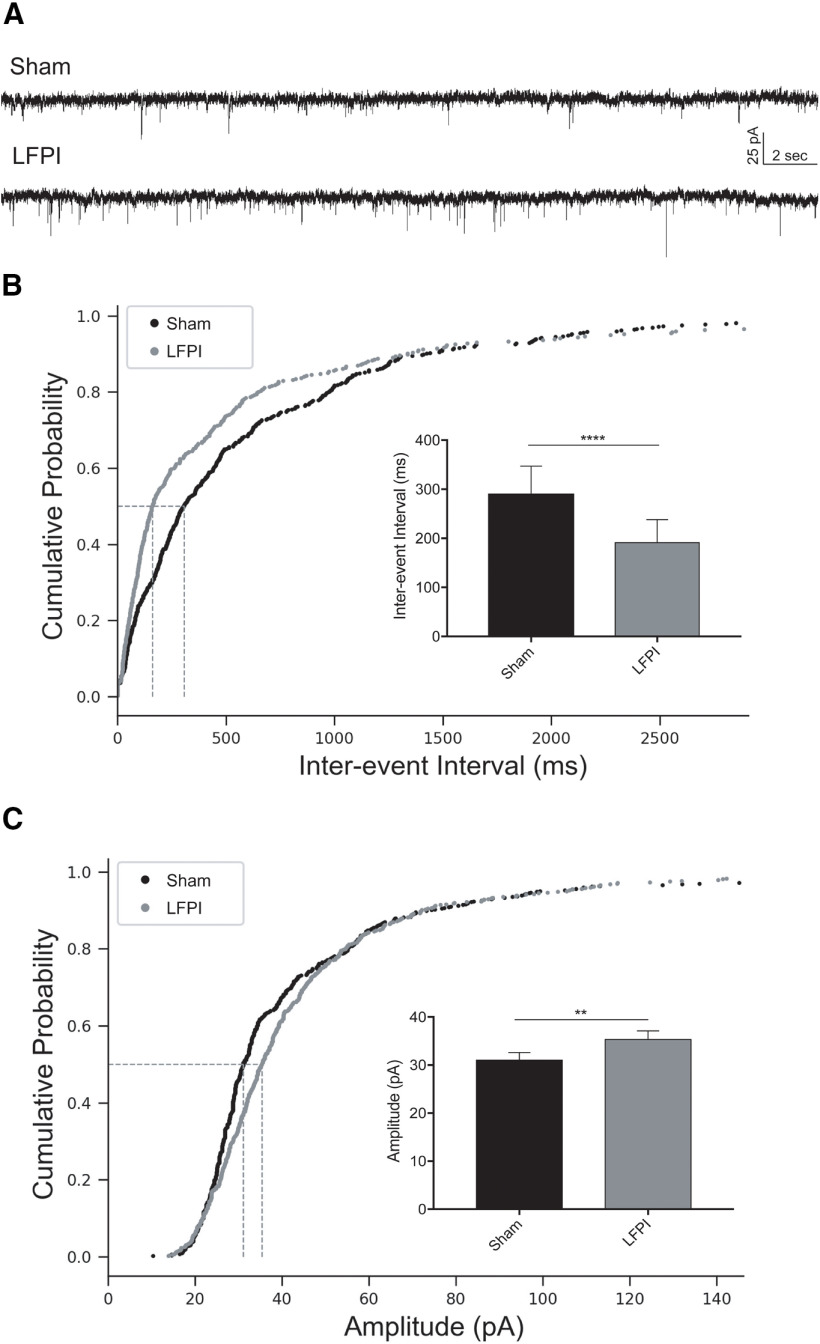
PV-IN mEPSCs are larger and more frequent after LFPI. ***A***, Representative traces of voltage-clamp recordings from sham (top trace) and LFPI (bottom trace) PV-IN cells in the presence of 30 μm BMI and 0.4 μm TTX to block presynaptic AP-dependent synaptic transmission. Cumulative probability plots of (***B***) mEPSC interevent interval and (***C***) event amplitude from sham PV-INs (black) and LFPI PV-INs (gray). Vertical dashed lines indicate the median of the distribution at probability, *p* = 0.5. Insets, Bar graphs of the median and 95% confidence intervals of (***B***) mEPSC interevent interval and (***C***) mEPSC amplitude in sham (black) and LFPI (gray) PV-INs; ****p < 0.0001, **p < 0.01.

To examine whether excitatory synaptic input to dentate PV-INs was altered after LFPI, whole-cell voltage-clamp recordings of mEPSCs were obtained from sham and LFPI brain slices (sham: *n* = 7 cells, 5 animals; LFPI: *n* = 6 cells, 6 animals). Representative recordings for each group are shown in Figure 5*A*. The frequency of mEPSCs was increased in PV-INs after injury as observed by a decrease in interevent intervals [sham: median Inter-quartile range (IQR) =281 (640.2) ms; LFPI: median (IQR) = 164 (436.9) ms, *p* < 0.0001, K-S test; Fig. 5*B*; Table 2, 4a]. Additionally, mEPSC event amplitudes were larger after LFPI [sham: median (IQR) = 30.8 (19.4) pA; LFPI: median (IQR) = 33.7 (19.9) pA, *p* = 0.006, K-S test; Fig. 5*C*; Table 2, 4b] and had faster rise kinetics [rise τ, sham: median (IQR) = 0.41 (0.70) ms; LFPI: median (IQR) = 0.32 (0.66) ms, *p* = 0.004, K-S test; Table 2, 4c]. No change in mEPSC decay kinetics was observed [decay τ, sham: median (IQR) = 0.99 (2.28) ms, LFPI: median (IQR) = 0.83 (1.92) ms, *p* = 0.20, K-S test; Table 2, 4d].

### PV-INs receive less feed-forward excitation from the perforant path after injury

**Figure 6.**
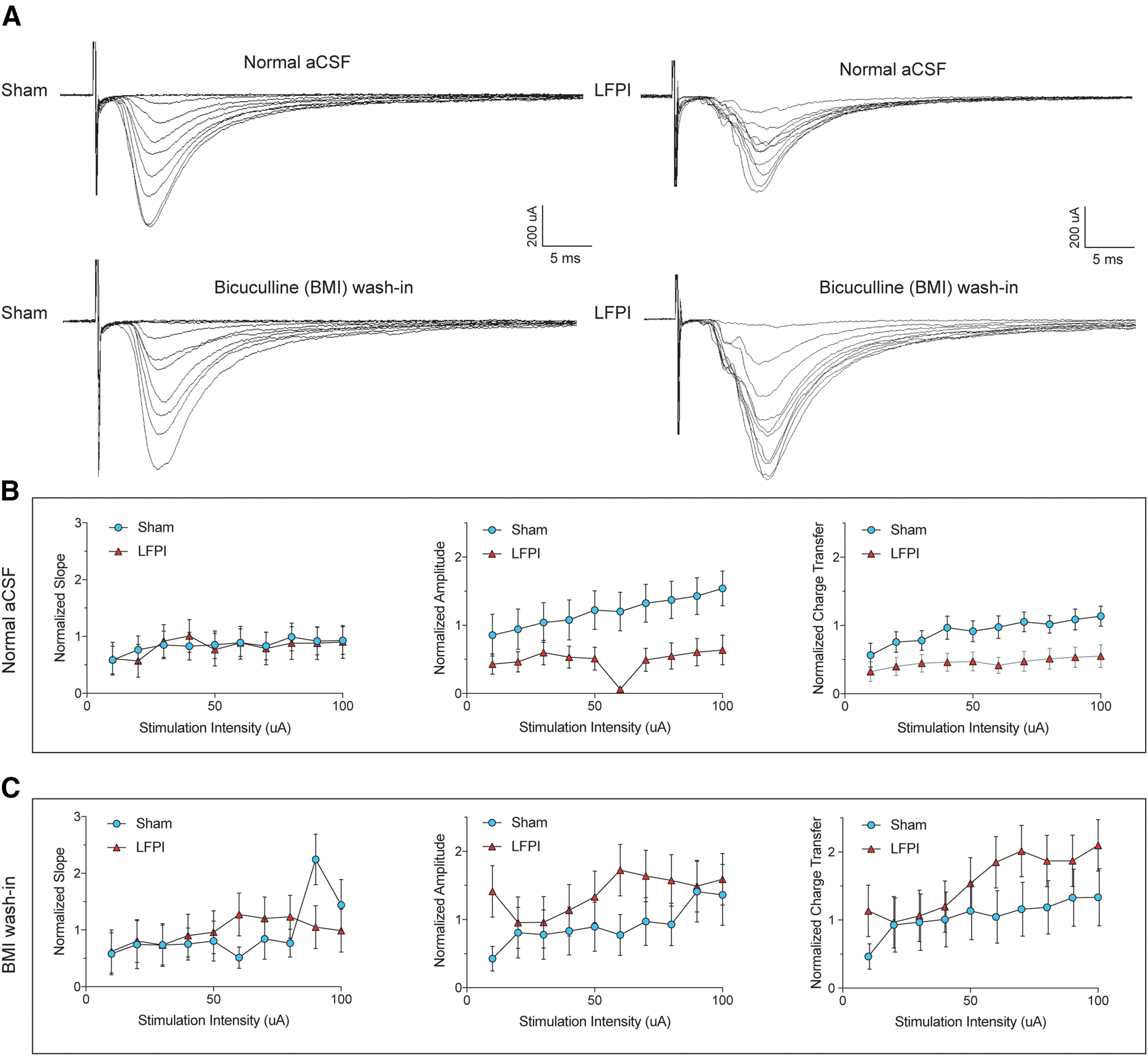
Decreased perforant path-eEPSCs onto PV-INs after LFPI. ***A***, Representative traces of perforant path-eEPSCs onto PV-INs from sham (top left), LFPI (top right), and the same sham (bottom left) and LFPI (bottom right) cells with the addition of BMI. eEPSCs were normalized to the fiber volley (FV) amplitude of the extracellular field response of that brain slice. The FV-normalized (***B***, left) slope, (***B***, middle) amplitude, and (***B***, right) charge transfer of eEPSCs in sham (blue) and LFPI (red) PV-INs. FV-normalized (***C***, left) slope, (***C***, middle) amplitude, and (***C***, right) charge transfer of eEPSCs after BMI wash-in.

Since local dentate glutamatergic neurons have demonstrated increased activity after experimental TBI (Lowenstein et al., 1992; Santhakumar et al., 2000; Gupta et al., 2012), it is likely that a decrease in AP-mediated excitatory drive is coming from perforant path synapses. To test the effect of injury on feed-forward excitation of PV-INs, we examined the I/O relationship of eEPSCs by electrical stimulation of the perforant path at incremental stimulus intensities (10–100 μA; Fig. 6*A*, top row). While the slope of perforant path eEPSCs was not significantly different between PV-INs from sham (*n* = 12 cells, 6 animals) and injured (*n* = 11 cells, 6 animals; repeated measures ANOVA, *F*_(1,13)_ = 0.1825, *p* = 0.68; Fig. 6*B*, left; Table 2, 5a), there was a decrease in eEPSC amplitude repeated measures ANOVA, amplitude: *F*_(1,22)_ = 4.715, *p* = 0.041; Fig. 6*B*, middle; Table 2, 5b) and charge transfer with increasing stimulus intensities (*F*_(1,21)_ = 5.426, *p* = 0.029; Fig. 6*B*, right; Table 2, 5c). BMI was then washed in to block local GABAergic inhibition onto cells in sham and LFPI slices, respectively, and evoked synaptic current properties were re-examined (Fig. 6*A*, bottom row). In the absence of inhibition, LFPI eEPSC I/O curves were similar to sham [eEPSC slope: *F*_(1,10)_ = 0.005, *p* = 0.94 (Fig. 6*C*, left); eEPSC amplitude: *F*_(1,10)_ = 0.86, *p* = 0.37 (Fig. 6*C*, middle); eEPSC charge transfer: *F*_(1,10)_ = 0.95, *p* = 0.35 (Fig. 6*C*, right); Table 2, 5d–5f]. There was no difference in the passive intrinsic properties of PV-INs used in current clamp experiments (resting membrane potential, sham: mean ± SEM = −64.5 ± 2.6 mV; LFPI: mean ± SEM = −61.0 ± 2.5 mV; whole-cell capacitance, sham: mean ± SEM = 43.4 ± 6.8 pF; LFPI: mean ± SEM = 43.7 ± 6.5 pF). These data suggest that diminished feed-forward excitatory synaptic transmission from the perforant path onto dentate PV-INs is because of GABAergic inhibition after injury.

### Increased synaptic inhibitory input onto PV-INs after LFPI

**Figure 7.**
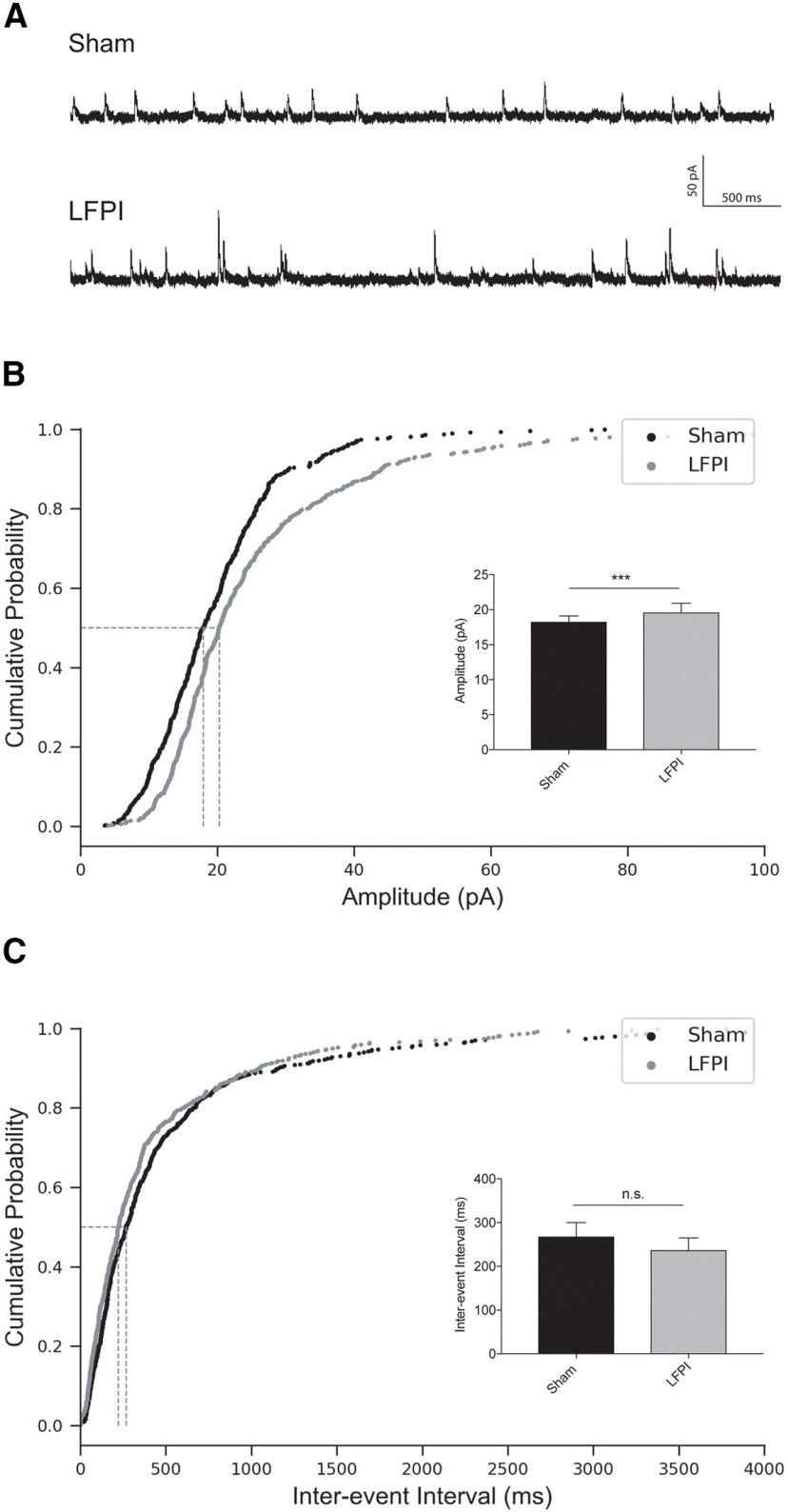
Increase in PV-IN mIPSC amplitude after LFPI. ***A***, Representative traces of voltage-clamp recordings at 0 mV from sham (top trace) and LFPI (bottom trace) PV-IN cells in the presence of glutamatergic blockers, APV and CNQX, and 0.4 μm TTX to isolate action-potential-independent synaptic transmission. Cumulative probability plots of (***B***) mIPSC amplitude, (***C***) interevent interval from sham PV-INs (black) and LFPI PV-INs (gray). Vertical dashed lines indicate the median of the distribution at probability, *p* = 0.5. Insets, Bar graphs of the median and 95% confidence intervals for respective event measurements in sham (black) and LFPI (gray). n.s., no significance; ***p < 0.001.

To understand how brain injury affects inhibitory synaptic transmission onto dentate PV-INs, we next recorded miniature AP-independent inhibitory events (mIPSCs; sham: *n* = 6 cells/animals, LFPI: *n* = 7 cells/animals; Fig. 7*A*). We observed an increase in the amplitude of mIPSCs after injury [sham: median (IQR) = 18.3 (11.6) pA, LFPI: median (IQR) = 19.6 (16.8) pA, *p* = 0.0003, K-S test; Fig. 7*B*; Table 2, 6a]. Rise kinetics were significantly faster in LFPI PV-INs [rise τ; sham: median (IQR) = 0.73 (0.71) ms; LFPI: median (IQR) = 0.63 (0.75) ms, *p* = 0.0079, K-S test; Table 2, 6b]. Injury did not affect mIPSC event frequency however, as the interevent interval between events was not significantly different between PV-INs from sham and injured slices [sham: median (IQR) = 268 (468.3) ms; LFPI: median (IQR) = (412.3) 237 pA, *p* = 0.26, K-S test; Fig. 7*C*; Table 2, 6c]. mIPSC decay τ also remained intact after injury [sham: median (IQR) = 5.6 (8.4) ms; LFPI: median (IQR) = 7.8 (9.7) ms, *p* = 0.86, K-S test; Table 2, 6d]. These results demonstrate that PV-INs receive larger AP-independent GABAergic events after LFPI, but that the frequency or ionotropic GABA_A_-receptor kinetics is unchanged.

### Decrease in PV-IN APs is because of net GABAergic inhibition

**Figure 8.**
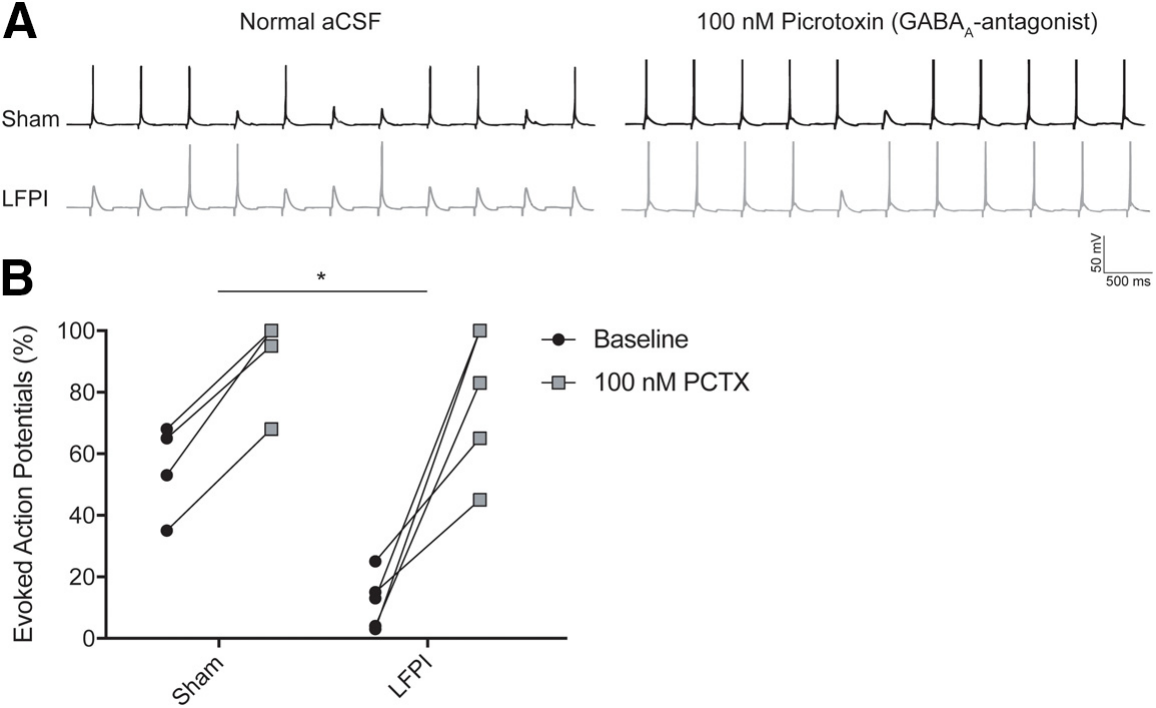
Feed-forward activation of PV-INs is diminished by increased network inhibitory input. ***A***, Minimal electrical stimulation evoked voltage responses for individual cells in sham (top left trace) and LFPI (bottom left trace) slices during whole-cell current-clamp recordings with a holding potential of −65 mV in normal ACSF solution (left) and with bath application of 100 nm PCTX (sham, right top trace; LFPI, right bottom trace) while recording the same cell with 12-s interstimulus intervals (intervals shorted to 500 ms in figure). ***B***, Percentage of evoked APs elicited in response to a sequence of 20 stimulations (i.e., 10 evoked APs in 20 stimulations = 50%) at baseline in normal ACSF perfusion solution and after bath application of PCTX. Percent differences in evoked responses for sham (mean ± SEM): 34.8 ± 3.1% (*n* = 5 cells), and for LFPI (mean ± SEM): 66.6 ± 13.31% (*n* = 5 cells); *p = 0.0008, Welch’s unpaired *t* test.

The results of the whole-cell patch clamp recordings indicate that there are changes in both excitatory and inhibitory synaptic inputs onto PV-INs after brain injury. These alterations affect the synaptic balance of PV-INs and influence the cells’ ability to fire an AP by depolarizing or hyperpolarizing the membrane potential. To understand the net effect of posttraumatic synaptic alterations on the feed-forward activation of PV-IN neurons, we performed a series of perforant path minimal stimulation experiments and counted the number of PV-INs evoked APs recorded in current-clamp (*n* = 5 cells, 5 animals, in each group; Fig. 8*A*). Before current clamp recording, resting membrane potential and whole-cell capacitance were measured in each cell. Neither resting membrane potential (sham: mean ± SEM = −64.5 ± 2.67 mV; LFPI: mean ± SEM = −61.0 ± 2.48 mV, *p* = 0.37) nor whole-cell capacitance [sham: mean ± SEM = 43.4 ± 6.81 pF; LFPI: mean ± SEM = 43.8 ± 6.48 mV, *p* = 0.64, Table 2, 7a,7b] differed between groups. In normal ACSF solution, PV-INs from LFPI slices had a significantly lower percentage of evoked APs than cells from sham slices (% evoked APs, sham: mean ± SEM = 57.8 ± 6.3%; LFPI: mean ± SEM = 12.0 ± 4.0%, *p* = 0.0008, Welch’s unpaired *t* test, Glass’s *d *=* *3.6; Fig. 8*B*, black circles; Table 2, 7c). There was no difference between the baseline stimulation levels of sham and LFPI groups (sham: mean ± SEM = 44 ± 21.6 μA; LFPI: mean ± SEM = 50 ± 21.7 μA, two-sample *t* test *t* = −0.19, *p* = 0.85). Next, 100 nm PCTX was bath applied to remove GABA_A_-mediated inhibition from PV-INs. PCTX significantly increased the percentage of evoked APs in both sham (sham pre-PCTX: mean ± SEM = 57.8 ± 6.3%; sham post-PCTX: mean ± SEM =92.6 ± 6.2%, *p* = 0.0004, paired *t* test, Table 2, 7d) and LFPI PV-INs (LFPI pre-PCTX: mean ± SEM = 12.0 ± 4.0%; LFPI post-PCTX: mean ± SEM = 78.6 ± 10.6%, *p* = 0.0034, paired *t* test, Table 2, 7e). After PCTX application, there was no difference in the percentage of PV-IN evoked APs in sham and LFPI groups (% evoked APs, sham: mean ± SEM = 92.6 ± 6.2%, LFPI: mean ± SEM = 78.6 ± 10.6%, *p* = 0.29, Welch’s unpaired *t* test, Glass’s *d *=* *1.13; Fig. 8*B*, gray squares; Table 2, 7f). These findings demonstrate that feed-forward activation of PV-INs is compromised by network inhibition.

## Discussion

In the DG, GABAergic basket cells and axo-axonic cells (i.e., PV-INs) are important drivers of feedforward inhibition onto granule cells (Kraushaar and Jonas, 2000; Ewell and Jones, 2010). Previous work has shown that feedforward inhibitory control of dentate granule cell firing is compromised after LFPI (Toth et al., 1997; Witgen et al., 2005). In seeking to identify potential cellular sources of granule cell disinhibition, this study is the first to demonstrate altered excitability of dentate PV-INs following experimental TBI. First, we showed that mild LFPI induced a loss of PV-INs in the hilus, recapitulating hilar interneuron loss observed in previous studies using moderate LFPI (Lowenstein et al., 1992; Toth et al., 1997; Santhakumar et al., 2000; Witgen et al., 2005). While surviving PV-INs have normal intrinsic membrane properties, excitatory and inhibitory synaptic currents are, respectively, shifted after injury. Furthermore, the data reveal that cortical feedforward activation of PV-INs is diminished because of a net inhibitory effect and lead to decreased evoked PV-IN firing. Together, our findings demonstrate a mechanism of reduced network inhibition contributing to DG and hippocampal dysfunction following TBI.

The observed hilar PV-IN cell loss demonstrates that interneurons in this subregion are vulnerable to cell death in a milder model of LFPI (1.4–1.6 atm). A recent study of mild LFPI in rats also found a non-specific decrease in PV-INs 7 d after injury (Zhang et al., 2018). The steady number of PV-INs in the subgranular zone and granule cell layers supports findings by Toth and colleagues, who proposed that laminar cell density plays a role in injury-induced neuronal loss, and with loose cell packing in the hilus leading to cell death susceptibility (Toth et al., 1997). An advantage of cell counts in the PV-Tomato transgenic mice used is that fluorescence protein expression is controlled by the *CAG* promoter and therefore was not directly linked to PV-expression after injury. Therefore, it is unlikely that the decrease in tdTomato-positive cell bodies is because of reduced PV protein expression or immunoreactivity (Nichols et al., 2018). However, we cannot rule out that brain injury may negatively affect CAG promotor activation and interfere with fluorescent protein expression.

When we examined the intrinsic membrane excitability of PV-INs, we observed no differences in passive or active properties following injury. Other dentate cells types have also been shown to retain their intrinsic properties, suggesting that the composition of membrane leak and voltage-gated channels are not overtly altered by LFPI (Howard et al., 2007; Santhakumar et al., 2000, 2001). This could reflect homeostatic compensation by opposing modifications of intrinsic currents, as was previously observed in mossy cells after injury (Howard et al., 2007). This is opposed to the transient depolarization found in dentate interneurons because of diminished Na^+^/K^+^ ATPase activity during the early acute postinjury period (i.e., 4 d after FPI; Ross and Soltesz, 2000). Therefore, at 7 d after injury, homeostatic mechanisms may have recalibrated PV-IN membrane properties. Further inspection of isolated ionic currents may be required to rule out the contribution of compensatory cellular processes of maintaining PV-IN intrinsic excitability and to better understand the dynamicity of cellular properties during the posttraumatic period.

While the intrinsic properties of PV-INs remained intact, changes in both excitatory and inhibitory synaptic currents reflected posttraumatic circuit-level alterations. On the excitatory side, mEPSCs had larger amplitude events, suggesting a larger postsynaptic response (e.g., increased insertion of receptors into the membrane) or larger presynaptic quantal size. The kinetics of mEPSCs were unchanged after injury. Several factors contribute to changes in rise and decay kinetics, including dendritic filtering, glutamate clearance, and receptor subunit composition. The lack of differences in the kinetics of mEPSCs between sham and LFPI groups suggests that factors like glutamate clearance and dendritic filtering may not play a prominent role in integration of EPSCs by PV-INs (Diamond and Jahr, 1997; Magee and Cook, 2000; Williams and Mitchell, 2008). Furthermore, PV-INs in LFPI animals demonstrated increased mEPSC frequency. More frequent mEPSC events may reflect in increased in basal excitatory transmission mediated by changes in release probability or increased presynaptic activity, the latter which has support from previous studies that local dentate glutamatergic neurons are more excitable after injury (Santhakumar et al., 2000; Gupta et al., 2012). This suggests that basal excitatory synaptic transmission onto PV-INs after LFPI may be a consequence of increased local glutamatergic activity in the DG network.

On the inhibitory side, mIPSCs demonstrated larger amplitudes but did not alter their frequency of occurrence, suggesting either an increase in postsynaptic response such as more GABA_A_-receptors inserted into the postsynaptic membrane, or the presynaptic packaging of larger GABA quantal sizes. Specifically, enhanced inhibitory events may indicate an increase in tonic GABA-receptor mediated currents, as have been shown in dentate PV-INs after status epilepticus and granule cells in a controlled cortical impact TBI model (Mtchedlishvili et al., 2010; Yu et al., 2013). Additionally, mIPSCs in LFPI animals had faster rise kinetics than in sham animals. The kinetics of IPSCs at GABAergic synapses are determined by the properties of their postsynaptic receptors. Differences in IPSC kinetics may suggest alterations in the activation or recruitment of GABA_A_-receptors with different subunit compositions. Phasic inhibition results are mediated by activation of synaptically located, α1 and γ2 subunit-containing postsynaptic receptors by saturating concentrations of GABA. Tonic inhibition results from activation of extrasynaptic GABA_A_-receptors containing α4 and δ subunit-containing by low concentrations of ambient GABA (Rossi and Hamann, 1998; Stell and Mody, 2002; Mody and Pearce, 2004). Extrasynaptic GABA_A_-receptors are slow to desensitize while their synaptic counterparts rapidly desensitize. Therefore, it is possible that faster mIPSC rise times suggest alterations in postsynaptic receptor subunit composition.

After LFPI, perforant path-eEPSCs were smaller than sham controls, but returned to sham levels when GABAergic inhibition was blocked by BMI wash-in. No change in eEPSC slopes indicated that the activation of the evoked response was not affected, but the amplitude and overall charge transfer are decreased because of an enhanced inhibitory tone onto PV-INs. Minimal stimulation experiments further demonstrated that net augmentation of GABAergic inhibition decreased PV-INs evoked APs in response to entorhinal afferent input. This finding has major implications for predicting the activity of PV-INs in the posttraumatic DG. It also provides additional evidence that injury-induced granule cell disinhibition may be attributed to decreased activation of feedforward GABAergic sources. While the results of our study do not provide a direct link between altered PV-IN firing and diminished GABAergic synaptic transmission onto granule cells, they do demonstrate network alterations in synaptic transmission that affect the functional activation of PV-INs. The posttraumatic decrease in mIPSC frequency onto granule cells seen previously suggests that there are fewer inhibitory synapses onto granule cells, potentially including loss of hilar PV-IN connections (Soltesz et al., 1995; Toth et al., 1997).

At the circuit level, loss of perisomatic inhibitory control of dentate granule cells is likely to disrupt the gating function of the DG after TBI. Additionally, decreased feedforward recruitment of PV-INs could have cognitive consequences. Fuchs and colleagues have previously demonstrated that loss of PV-IN recruitment lead to impaired performance on hippocampal-dependent behavioral task in mice when excitatory drive onto PV-INs was knocked out (Fuchs et al., 2007). Therefore, alterations in PV-IN activation and network recruitment could have profound effects on cognitive processes and potentially underlie hippocampal-dependent cognitive deficits experienced by TBI patients. Future studies would benefit from investigating potential changes in the properties of PV-IN synapses onto granule cells, as previous work suggests that these synapses may have higher failure rates and smaller pools of readily releasable vesicles in a model of dentate network hyperexcitability (Zhang and Buckmaster, 2009).

There are several limitations of this study. It is important to note that perisomatic inhibitory control of dentate granule cells is provided by nonoverlapping populations of PV+ and CCK^+^ basket and axo-axonic cells (Freund and Buzsáki, 1996; Soriano et al., 1990). While this study only examined PV-INs after TBI, understanding the effects of CCK^+^ basket cell inhibition will provide a complete picture of alterations in granule cell perisomatic inhibition. In addition, we did not investigate possible mechanisms of synaptic alterations such as the number of synapses, presynaptic probability of release, or firing activity of presynaptic neurons. Future investigation of the mechanisms of excitatory-inhibitory (E/I) imbalance are crucial to understanding the overall shift in network excitability. Lastly, while both male and female mice were used for experiments, we did not explicitly explore sex as an experimental variable in this study. Previously, our laboratory has demonstrated that female and male mice similarly experience higher fEPSP I/O curves 7 d after LFPI (citation redacted), however the current study does not explicitly compare sex differences in dentate PV-IN physiology after injury.

In conclusion, our data show that synaptic input onto dentate PV-INs is altered after injury and is associated with diminished afferent activation of PV-INs driven by network inhibition. Inhibition decreases AP initiation and suggests that activation of PV-IN-mediated feedforward inhibition onto granule cells in the DG is compromised following brain injury. These results demonstrate posttraumatic alterations in inhibitory function that may contribute to dentate network hyperexcitability and may hold therapeutic significance in the future as a specific cellular target for restoring hippocampal dysfunction after TBI.

## Synthesis

Reviewing Editor: Christophe Bernard, INSERM & Institut de Neurosciences des Systèmes Decisions are customarily a result of the Reviewing Editor and the peer reviewers coming together and discussing their recommendations until a consensus is reached. When revisions are invited, a fact-based synthesis statement explaining their decision and outlining what is needed to prepare a revision will be listed below.

Dear authors,

Since your original reviewing editor rotated off, I stepped in. With you original reviewers, we agree that the manuscript has been improved and that you are addressing an important question in TBI research: the fate of PV+ neurons.

Some major concerns remain.

The method section does not provide enough details.

a) How did you build the cumulative distribution functions? It is important to use the exact same number of events (say 50-100) per cell to guarantee that the properties found in one cell do not bias the overall distribution. Fig. 6B is a typical example. The LFPI curve crosses the sham one. Hence, in LFPI conditions there are more events with short inter-event intervals and more events with long intervals. Is it true for every recorded cell, or is due to a cell or two which bias the distribution?

b) How did you calculate the slope of fEPSPs and EPSCs? Figs 4 and 7 show polysynaptic events. The slope of the monosynaptic part (20-80% rise time) should be calculated.

We are puzzled by the way the electrophysiological study was conducted. Since you used a CsGlu solutions you could have recorded sEPSCs, sIPSCs, mEPSCs and mIPSCs in the same cells, without using antagonists. This would have allowed you to calculate the E/I ratio in each recorded cell for both spontaneous and miniature currents. This is the standard procedure used in numerous studies. The use of antagonists and measuring E and I in difference cells renders the interpretation very complicated, as you are driving the network in a non-physiological regime. The best example is brought about by the the m/s EPSC data. In LFPI animals, the spontaneous events go from a mean of ˜350ms (Fig 5D) to just under 200 ms for miniature events (Fig 6B). That means, the frequency of EPSCs nearly doubled after putting TTX on the slice. We simply don’t understand how this can be. Perhaps when you block inhibition, excitatory cells are more active release more glutamate, leading to the activation of metabotropic receptors, which would decrease glutamate release on PV cells. Activation of CB1 receptors, GABAB receptors and many others would do the same. After TTX, the activity-dependent effect would disappear. The only interpretable conclusions are those from miniature recordings. Hence, we request that you remove any claim/conclusion about E/I imbalance, simply because the experimental protocol does not allow you to evaluate it. You may consider removing the analysis of spontaneous currents. Alternatively, if you wish to keep it, you must discuss the caveats of the use of antagonists.

Other major concerns:

1. The PV immunostaining in Fig 2 does not resemble what is expected of PV staining in the dentate. Typically, there are a few cells and dendrites in the molecular layer and hilus with a mesh of axonal staining in GCL (see DOI: 10.1126/science.1255263)..this seems to be the case in the tdTomato. The dense molecular and hilar staining with the PV antibody used in the study suggests that the PV antibody used here may lack specificity.

2. Line 305: The use of Two-Way ANOVA to test for effect of stimulus evoked response in sham vs. LFPI is puzzling. There is only one independent variable (sham vs LFPI) and multiple levels of stimulus tested - shouldn’t this be Repeated measures ANOVA?

3. Line 314: the term interneurons for mossy cells and semilunar granule cells is not appropriate as these are not local circuit neurons. Mossy cells project contralaterally and semilunar granule cells project to CA3. The term interneuron, while traditionally used to denote local circuit neurons (semilunar granule cells are projection neurons) is more often used to refer to GABAergic neurons and rewording to indicate these are glutamatergic neurons would avoid confusion.

4. The minimal stimulation experiments has been improved, but they are still of particular concern/confusion: Since the stim intensity was set for each cell (lines 237-239), as is appropriate, there is a possibility of systematic difference in the stimulus intensity needed to elicit action potential in Sham vs. LFPI. If as indicated the stim intensity was tuned to elicit one stim for 20 stimuli, it is confusing that the success rate is different between the two groups. Information on the differences in basal stimulation need to be included. Regardless, there are really three results in these data. First, in normal ACSF, the percentage of APs evoked by perforant path stimulation is significantly lower in LFPI slices compared to controls, as described. Second, it appears that PTX application significantly increases the percentage of evoked APs in both control and brain injured slices. This should be analyzed by a paired two sample test and reported. Did you confirm that 100nm PTX eliminated IPSCs? Third, in PTX, you state that there is no difference in % of evoked APs. However, the responses in LFPI cells is quite variable. A sample size of N=5 is too small for the approximate power calculation to be reliable, and post-hoc power analyses are not as meaningful as evaluating effect sizes.

5. Lines 535-549: Relevant literature suggesting changes in tonic GABA currents in dentate PV neurons in disease (doi: 10.1152/jn.00891.2012) should be considered

6. The graphical abstract is confusing. If the intent is to show an increase in inhibition and decrease in excitation on the scales are tipped the wrong way. The item that is lower on a scale is heavier. If the intent is to show EI balance on PV neurons, the red and green dots need to be flipped. If the intent is to show effect on the dentate networks, the relevant data on Es is not in this manuscript. Overall, the scale is confusing and needs to be removed/revised.

Minor concerns:

1. Multiple typos in abstract with “perforant” path autocorrected to performant path.

2. Line 9: “Evoked EPSC in PV-INs” should be “Evoked EPSCs in PV-INs” ; and Lines 11-12: current clamp recordings ... cannot decrease a propensity.

3. Line 13 reads “after LFPI and could be...” Consider instead: “after LFPI, which could be...”

4. Lines 25-26 reads “The disinhibition of PV -INs...” when the previous sentence said that there is decreased firing of PV-INs, therefore they’re not disinhibited. Did you mean to say “The inhibition of PV-INs...” or “disinhibition of dentate”?

5. Line 115 reads “in dentate gyrus (Figure).” Did you mean “in dentate gyrus (Figure 1).”?

6. Line 173-174: “were recording in response to” should be “were recorded in response to"

7. Line 253: introduce the acronym “(K-S)” after the first use of Kolmogorov-Smirnov test

8. Line 460: the use of “resultantly” is odd and needs to be revised

9. Lines 486-487: “Further inspection isolated ionic currents may be required” should read “Further inspection of isolated ionic currents may be required"

10. Line 522: “activity is increased onto PV-INs after injury” better as “ activity onto PV-INs is increased after injury"

11. Lines 535-536: consider rephrasing to improve grammar and clarity: “Phasic inhibition results in mediated by activation of synaptically located, alpha1 and gamma2 subunit-containing postsynaptic receptors by saturating concentrations of GABA.” Did you mean “results from activation of...” or “results are mediated by activation of...”?

12. Line 542: “while miniature events many reflect activation of extrasynaptic receptors” should read “while miniature events may reflect activation of extrasynaptic receptors"

13. Lines 740-741 should clarify that the asterisk in C and F indicates the location of the inset.

14. Line 835 reads “Shame, right top trace” when it should say “Sham, right top trace"

15. Figure 3 legend should specify that F is an inset of E. Maybe draw a box around the area in E that is blown up in F and specify that in the legend.

16.Figure 7B and 7C lack units on the y-axis. Figure 7A does not need to have “Bicuculline (BMI)” spelled out (can just say “BMI”) when it’s defined as “bicuculline methiodide (BMI)” in the legend.

17. Figure legend for Fig 8 says “Shame” when it should read “Sham”.
